# Free will debates: Simple experiments are not so simple

**DOI:** 10.2478/v10053-008-0076-2

**Published:** 2010-08-30

**Authors:** W. R. Klemm

**Affiliations:** ^1^College of Veterinary Medicine & Biomedical Sciences, Texas A&M University, Texas

**Keywords:** free will, consciousness, Libet, compatibilism

## Abstract

The notion that free will is an illusion has achieved such wide acceptance among philosophers and neuroscientists that it seems to be acquiring the status of dogma. Nonetheless, research in this area continues, and this review offers a new analysis of the design limitations and data interpretations of free-will experiments. This review presents 12 categories of questionable conclusions that some scholars use to promote the idea that free will is an illusion. The next generation of less ambiguous experiments is proposed.

## INTRODUCTION

Most non-academics tend to take as a given that people can freely make decisions and choices when there are alternatives and absence of external constraints. A case for consciousness causing certain behaviors has been made by Pockett, Banks, and Gallagher (2009). Nonetheless, there is a growing body of scientists and philosophers, many of whom are acknowledged as scholars of the first rank who acknowledge consciousness as a distinct mental state, yet conclude that free will is an illusion, a trick played on us by the brain. This view dates back for hundreds of years, but in our time the debate has intensi fied, in large part because of what I think is misinterpreted research.

The purpose of this review is to incorporate the findings of recent research into the evolving understanding of the enduring scientific and philosophical controversy over whether humans have free will. This review identifies 12 categories of concerns that are, in the author’s view, not adequately considered by those who argue that free will is an illusion. This review also provides some suggestions for improving the design of future experiments.

Analysis of the controversy requires clear definitions of a few terms, which unfortunately are often used colloquially with poor precision. To a degree, such problems are inevitable. Nonetheless, operational definitions are helpful. *Free will* could be defined in various ways. *Will* is herein operationally defined here by such synonyms as *intent*, *choice*, or *decision*, and it can be accomplished consciously or subconsciously. *Free* implies a conscious causation in which an intent, choice, or decision is made among alternatives that are more or less possible of accomplishment and are not constrained by either external or internal imperatives for the embodied brain.

It seems important to emphasize that not all conscious actions are freely willed. One is often consciously aware of one‘s actions that may have been subconsciously generated, as in observing one’s own knee-jerk reflex. So one task of free-will research is to design tests that distinguish conscious awareness from conscious choice (free will).

Defining *consciousness* is much more problematic. Pacherie (2009) suggests there are two ways to think about consciousness: The first idea is that consciousness is a state where one is conscious (aware?) of an object, property, or state of affairs. This strikes me as a circular definition, which can also be found in many dictionary definitions. The second aspect of consciousness is that it is a state where one “has a representation of that state as a specific attitude toward a certain object, you are aware. This is perhaps easiest to comprehend if conscious ness is regarded as a neurophysiological avatar, generated as a neural representation of self, aware of events in the environment in the context of itself. Such an avatar could be a self-aware active agent of the embodied brain, an argument that I pursue in another manuscript.

## The zombie argument

Those who argue against free will arrive at their counter-intuitive conclusion from research that does seem to challenge the traditional common-sense view of free will. *Zombian* is used as a semantic shorthand to describe those who subscribe to some form of Thomas Huxley’s view that humans are “conscious automata” whose brains cause behavior without conscious intent. Charles Darwin and Albert Einstein had also voiced similar zombian points of view.

Some very prominent modern scholars have expressed sympathy for the zombian view of human existence: Daniel Dennett, Patricia Churchland, Marc Jeannerod, Michael Gazzaniga, Hakwan Lau, Benjamin Libet, Henrik Walter, and Daniel Wegner.

The zombian idea has been tested in several formal studies that attempt to show that intentions are generated subconsciously – that is, free will is considered an illusion. Consciousness can only produce awareness of intentions; it can’t cause anything. Some zombians concede that consciousness can veto certain subconscious decisions. A role for conscious choice in programming the subconscious is seldom considered in these debates.

People with brain injuries provided the first arguments against free will. For example, people with injuries that caused amnesia were studied by British psychologists, Elizabeth Warrington and Lawrence Weiskrantz (1968). They showed a series of words to the amnesics, who could not remember the words. Then the patients were shown the first three letters of each word and asked to complete the letters to make a word, any word. Amazingly, they consistently conjured a word that was exactly the same as the one they had just seen and forgotten. In other words, the words were memorized in the subconscious mind but not the conscious mind. But this could just indicate a memory recall problem. What has this got to do with intentions?

The zombian argument may have begun catching on with the book by Julian Jaynes, *The Origin of Consciousness and the Breakdown of the Bicameral Mind* (1976). Jaynes gave many logical arguments that consciousness is not necessary for thinking and that most human mental work is done subconsciously, only becoming realized consciously after the fact. Jaynes concluded that consciousness is used only to prepare for thought and to perceive and analyze the end result of thinking. Experimental evidence was not provided.

Subsequent zombian theorists argue that decisions are made subconsciously and the conscious mind lays claim to them as its own. This position holds that the brain is an automaton that creates its own rules and makes sure that we live by them. The brain is in charge of itself.

Zombian theorists argue that human personality and behavior are predetermined and predictable, controlled by genetics and by how the brain has been programmed by the social and physical environment. There is no recognition that conscious mind can program the subconscious, as in learning to play the piano or riding a bicycle, for example.

Zombians cite the existence of compulsions and addictions as examples where conscious awareness fails to control the brain. The conscious mind knows when we have bad behaviors but can’t do anything about it. Our excuse is that we are addicted, have a brain disorder, or have been programmed by bad events beyond our control. The same kind of logic is used to explain character or personality flaws. We say, for example, “He can’t help it. That’s just the way he is.” Or “She really doesn’t mean to be that way.” Or “I can’t believe he did that. He is such a good boy.”

A more complete philosophical argument is provided by Henrik Walter (2001). He says our standard theory of mind is wrong, a mere convenience that satisfies our expectations about what people do. Walter says that criminals cannot be held responsible for their crimes. He argues that the correct notion is that we are automatons, albeit ones that are aware of what we are automatically doing. I think Walter is saying that conscious mind is only partly aware of the choices made by the subconscious. Conscious mind can only “look in” on what the *real* mind is doing. At best, a common view is that conscious mind can only monitor and perhaps veto choices made subconsciously. A more liberal elaboration is that free will operates “to ensure the continuity of subjective experience across actions which are – of necessity – executed automatically” (Jeannerod, 2009, p. 37).

A complete defense of the zombian school of thought is in the book by Daniel Wegner (2002). Leading thinkers, such as the philosopher, Patricia Churchland (2002), and the neuroscientist, Michael Gazzaniga (1998), recognize the nihilistic nature of the zombian conclusion but are resigned to a position of “it must be so.”

The most recent book perpetuates the zombian argument at least for many short-term intentions and asserts that the question remains open for all other intentions (Pockett et al., 2009).

Philosophers seem to polarize around two points of view: People lack free will but sometimes may have it (compatibilism) or human thoughts are beyond personal control and incompatible with free will (incompatibilist, i.e., “zombian”).

Some kind of logical reconciliation seems needed, and this is what gives urgency to the compromise of compatibilism. Most contemporary philosophers seem to hold the compatibilist view, namely that human beliefs and actions arise from a subconscious zombie-like mind, but it is wrong to assert that humans have no element of free will. Since free will is necessary for moral responsibility, one either has to accept free will or reject the notion that humans are responsible for what they believe and do.

In modern times, the free-will conundrum has been exacerbated by neuroscientific evidence that seems to conflict with the notion that people are responsible for their beliefs and actions. The accumulation of experimental evidence began with the simple experiment performed and elaborated in the 1980s by University of California scientist, Benjamin Libet. Thus, this present analysis will focus on the prototypical Libet experiment and those of others that followed in order to identify its strengths and weaknesses concerning the issue of free will.

I wish to focus on recent neuroscience that has aroused the passions of scholars and provided evidence that confuses the issues. Hopefully, I can provide some comfort to those neuroscientists who feel intimidated by philosophical sophistry into believing their data supports determinism at the expense of free will, when they might have thought their experiments were simple and easily interpreted. Here, I argue that zombian interpretations are based on flimsy evidence and specious arguments.

## A New Critique of Zombian Research

My critique will focus on three methodological arguments: (a) timing of when a free will event occurred is not accurately identified by introspection, which has been the dominant paradigm in zombian research, (b) free-will events are not readily captured by many of the kinds of tasks and procedures that have been used in zombian research, and(c) neurophysiological measures have been inadequate.

Two main lines of research provide the scientific underpinnings for modern zombianism. One is the paradigm developed by Wegner in the 1990s in which subjects were asked to move a cursor randomly around a computer screen and stop the cursor every 30 s or so over an object depicted on the screen (see e.g., Wegner, 2002). After each stop, the subject introspectively rated their intentionality in terms of how sure they were that they made a conscious decision to stop the cursor or whether the experimenter had made the manipulation behind the scenes. Subjects were quite bad in making such estimations. They were correct only 56% of the time that they had actually caused all of the stops. Wegner developed a later approach by having subjects view other people’s gloved hands located in the position where their own hands would be. As the gloved hands performed actions, subjects were asked to rate the extent to which they had controlled the movements. Again, subjects performed poorly in such estimates.

Wegner showed that the conscious sense of voluntary control increased when conscious prior thoughts corresponded to observed actions. From this, Wegner inferred that free will was retrospectively inferred. However, such results do not seem to provide unequivocal evidence against free will. Is it not possible that, regardless of accuracy, subjects had a pre-existing free will decision to stop the cursor whenever they wanted to? Is it not possible that their inaccuracies in assessing voluntary control arise from incomplete information and the inherent uncertainties in the task?

Just as the zombian conclusion of Warrington and Weiskrantz (1968) is suspect because their experiment measures memory recall more than conscious intent, Wegner’s conclusions are not compelling, because his experimental designs seems to test more than free will. My objection to the design is that one cannot conclude unequivocally that the intent is either conscious or subconscious, and that the major uncontrolled variable is the level of reliability of the subjects’ awareness of their conscious intent. Tim Bayne (2009) has written a more exhaustive criticism of Wegner’s zombianism based on the extreme complexity of the experience of conscious will.

The second line of research providing the scientific underpinnings for modern zombianism is the Libet Experiments. Libet (1985) monitored a “voluntary” finger movement while at the same time recording brain waves from the scalp overlying the part of the brain cortex that issues movement commands to the fingers. Participants were asked to make a spontaneous finger movement, at a time of their choice, while watching an electronic spot moving around a clock face. Subjects were to note the time on the clock at the instant that they decided to move the finger. When subjects consciously decided to make a movement, they reported the time of the decision from watching the modified clock. As expected, subjects thought that they had decided to move about a half second before actual movement, which is consistent with the idea that they willed the movement to occur.

**Figure 1.**
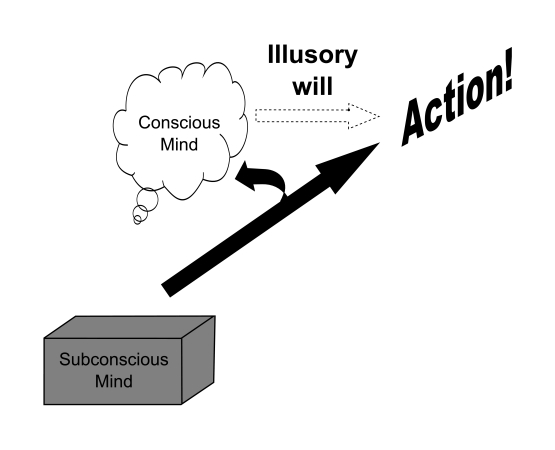
The concept of free will as an illusion. Subconscious mind is said to create behavior and belatedly lets conscious mind aware of what has already been done. T. H. Huxley called conscious will to act as a mere “symbol” of the processes that ge-nerate action.

But was that willed action “free?” The startling finding was that a major change in the EEG signal from motor cortex was observed about 350 ms before the subjects claimed that they willed the command to move. This EEG signal, discovered many years ago by others and dubbed “readiness potential,” was chosen by Libet to index the moment of decision. One interpretation of such a result, is that the decision was made unconsciously and consciousness is not part of the cause. Accepting that premise, one is forced to conclude that one does not “will” such movement, but merely retrospectively confirms that there was a willed action which must have been developed subconsciously. The brain just subconsciously decides to move and lets the conscious mind know what it has decided. The disturbing corollary is that one does not freely “choose” to do anything. The brain is just driven by external and internal forces to direct behavior, and one’s consciousness is only around to know about it (see Figure 1).

The Libet-type study relies on introspection, and the consensus of investigators subsequently using similar paradigms has been that participants were correctly aware of the time at which they thought they made the decision. The insufficiently addressed problem is the reliability of both introspection and accuracy of timing awareness.

Libet claims that humans cannot consciously initiate a choice, because the motor cortex “readiness potential” begins to develop 400 ms before a subject is consciously aware of an intent to act. But, since awareness of intention occurs 150 ms before actual movement, it is possible that one can freely choose to veto or inhibit an act that is triggered by subconscious command. He even demonstrated that subjects could veto their readiness potentials. Libet also took great care to rule out a role for misperceived timing, but I will argue later that such introspection cannot be reliable.

The Libet-type study also relies on a limited set of neurophysiological data. The premise is that monitoring a small piece of brain, such as the motor cortex, can serve as the indicator for conscious decision. Surely, there must be electrical indicators of conscious decision-making somewhere else in the brain, and it may have preceded development of the readiness potential. More recent investigators have indeed documented increased brain activity prior to the increased motor cortex activity, and these include areas not normally associated with movement (see below). Nobody knows where in the brain the conscious self is, much less where intentions are first initiated.

Another problem: The part of the cortex that was monitored, the motor cortex, only began its increased activity before the self reported intent to move. Few analysts admit how little we really know about what is signaled by this “readiness potential.” This will be explored in some depth later in this paper.

### Follow-up studies

In a follow-up to the Libet experiment, human brain scans were taken as subjects were asked to report when they first felt the urge or intention to move (Lau, Rogers, & Passingham, 2004). The brain scan images showed three small cortical regions of activation when the subjects attended to the urge to move prior to the actual movement itself, about 0.25 s before the actual movement, which is consistent with Libet’s results. But conscious intention was associated with increased neural activity in areas other than the motor cortex. These activations could well occur before the motor cortex is activated, but the imaging method used does not have the time resolution to answer this question. But even these limited results show that limiting analyses to the motor cortex is not sufficient. This is reinforced by the findings of Obhi and Haggard (2004) who found that awareness of conscious intent correlates more specifically with a motor cortex potential over the side of the head opposite to the hand making the movement (hand movements are initiated from the opposite cerebral hemisphere).

A follow-up study by the Lau group did examine more closely the timing judgment issue (Lau, Rogers, & Passingham, 2006). Specifically, they examined Libet’s finding that subjects misestimated the onset of movement, thinking it occurred about 50 ms before it actually did. In this Lau study, participants watched a red dot revolving around an unnumbered but calibrated clock face and introspectively indicated where the dot was when a conscious decision was made. They were required to fixate their gaze at a cross presented in the middle of the clock face, and press a button with their left index finger at a random time (whenever they felt the urge) after the dot has finished the first revolution. After the button was pressed, the dot disappeared after a variable period of 1280–2560 ms. A random variable delay was used so that subjects could not use the point where the dot disappeared to infer when they had pressed the button. After an additional 4–10 s variable delay period, the red dot appeared again at the middle of the clock. The participants used a game-pad with their right thumb to control the dot on the screen as a cursor. They were required to move the dot to where it was on the clock face when they pressed the button. After the cursor stayed still for 1 s, it disappeared, and the position of the dot was recorded by the computer, and the difference in the recorded position and the position during the onset of the button press was translated into milliseconds. In the “action nontiming” condition, the dot disappeared after revolving for one cycle. The participants were required, as in the action timing condition, to make a spontaneous button press. When the button was pressed, the red dot reappeared briefly (duration: 200 ms) at a random location around the clock face and the participants were required to remember this location, which they had to report after a 4–10 s variable delay using the same method as in the action timing condition. While this is a significant departure in monitoring timing of events from the original studies, it still involves introspectively deciding when an action was willed and in addition introduces complex cognitive variables.

The Lau group reasoned that there must be some place in the brain that signals the judgment that movement has occurred and that across subjects the magnitude of the brain activity correlate would positively correspond to the accuracy of the time estimate. Alternatively, enhanced electrical activity might contribute to the time-estimate error, in which case the correlation would be negative. They also re-examined their earlier fMRI data to see if the same principle applies for judgment of the onset of intentions.

What they found confirmed many earlier studies that indicated that the brain makes errors in time estimation. When participants were required to estimate the time onset of their movements (instead of their intentions), the activity in the cingulate motor area was enhanced. Moreover, across subjects the level of cingulate activity was positively correlated with time-estimate accuracy. That is, the greater the cingulate activity, the earlier subjects estimated the time of movement. The same principle seems to hold true for their earlier data on time estimates of onset of intention, as indicated by MRI changes in the pre-supplemental motor area. In other words, in both cases, time estimation could not be relied upon as accurate.

The recent studies by Chun Soon and colleagues (Soon, Brass, Heinze, & Haynes, 2008) used brain imaging in a design that was akin to Libet’s. However, they used a different method for introspective estimation of the instant of conscious decision. Subjects were asked to fixate on a screen where a stream of letters was presented. When they felt the urge, they were to decide on pressing one or two buttons, operated by right and left index fingers. At the time of button press, they were to register and remember the letter that was present at the time of decision. After the button press, they were presented with a screen that had four letters on it, and subjects indicated which one was present at the time of decision.

Another difference from the Libet studies was that more than one behavioral option was required (press left or right). This was intended to counter the argument that Libet’s observed anticipatory electrical change might have reflected some kind of nonspecific preparatory activation.

They monitored the same area as did Libet, the supplemental motor area of cortex (SMA). However, they reasoned that the SMA is active in the late stages of a movement decision, and that other brain areas might be involved in movement planning at earlier times.

**Figure 2.**
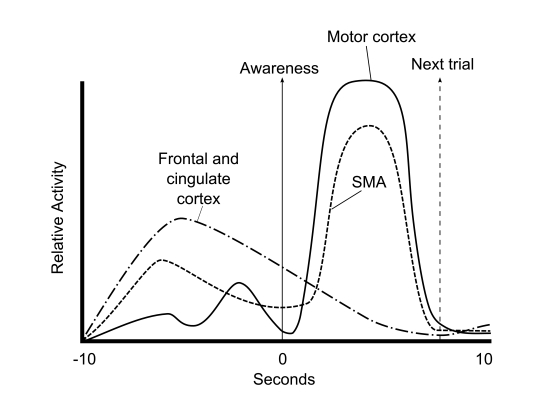
Change in MRI activity in supplemental motor area (SMA), motor cortex, and localized areas in the frontal and cingulate cortex, before, during, and after awareness of a freely determined decision to press either a left or a right button. Graphs are re-drawn for illustrative purposes to show the timing relationships between the awareness of decision and MRIRI activity in the respective brain areas. From the data of C. S. Soon, M. Brass, H.-J. Heinze, & J.-D. Haynes, 2008, “Unconscious Determinants of Free Decisions in the Human Brain,” *Nature Neuroscience, 11*, 543-545.

What they found was astonishing (see Figure 2). Two regions in the frontal and cingulate cortex exhibited a decision-predictive change a full seven-to-ten seconds before conscious awareness of the decision. The areas of the motor cortex that actually issue movement commands showed slightly increased activity a second or so prior to the instant of decision, and much more pronounced activity about 2 s after the decision. The antecedent activity was seen only in the right motor cortex of presumably right-handed subjects, a point that the authors chose not to interpret.

Activity in brain areas directly involved in issuing movement commands (SMA and motor cortex) increased greatly after decision. Increased activity in the other areas prior to awareness can be interpreted in more than one way. Most people, especially the lay press, assume that these other areas are subconsciously processing the decision to move and thus indicate absence of free will because they occur before subjects think they willed a movement. The authors were more restrained in wording their conclusion; namely, that the frontal and parietal cortical areas “influenced” the decision making up to 10 s before conscious decision to press one of the two buttons was realized. They view this early, pre-conscious activity as preparatory and also as a specific predictor of which button was to be pressed, but they did not choose to speculate further.

To me, an obvious interpretation is that frontal and cingulate cortex could have been processing the “rules of the game” and the free-will intent to move. The overlap with SMA activity seems inevitable in that rules of the game form a conscious context in which a willed act could occur at any moment. Obviously, rules of the game have to be processed initially in consciousness. However, once well-rehearsed, implementing intentions may be done without conscious awareness. However, a recent test of this issue by Bongers, Dijksterhuis, and Spears (2010) revealed that people do become aware of their goals and intents when pursuing a complex goal. It remains an open question whether this might apply to the Soon studies.

This study has the same limitations as the others of presupposing that the decision to move and the conscious realization are instantaneous. As with the original Libet experiments, experimenters relied on self-report of the decision to move, which no doubt has limited time resolution and accuracy. This design is, however, better in that subjects recalled what letter was being viewed on the screen at the “instant” of decision. Their methods allowed looking back further in time prior to the “instant” of decision and in evaluating other brain areas that might be involved in the movement planning process.

In designs like this, the subject knows as soon as one trial is over that another is beginning. Moreover, the subject consciously chooses to make a movement and the brain no doubt is planning to make such a movement long before a “go” signal is delivered via any decision-making process. So, the pre-movement increased brain activity could actually reflect conscious processing in working memory of the “rules of the game” and the *will* to obey those rules. It is true that humans are not necessarily aware of all contents of working memory (Cowan, 1997). But, all through a trial such as this, before decisions are made, the brain could be consciously processing in working memory a significant portion of at least five different things: (a) “I will make a button-press movement,” (b) “I will make the press either on the right or left,” (c) “I will notice the letters on the screen and hold them in working memory,” (d) “I will issue a *go* decision voluntarily,” and (e) I will remember which letter was present on the screen when the *go* command is issued.” Accordingly, there may not be any single electrophysiological marker of when a decision is made, conscious or otherwise. This study could actually support a free-will interpretation. The activity increase in non-motor areas could have reflected conscious decision making before the actual movement. In other words, the “go” decision was only one final part of the consciously willed process.

A more recent study was that of Michel Desmurget and colleagues (2009) in France, who took a different approach. First, they distinguished between two processes, the will to make movements and the awareness of such willed action. This led them to consider the parietal cortex as a possible site that brings intentions into conscious awareness. Secondly, they used direct electrical stimulation rather than recording. The subjects were awake humans with electrodes inserted into the brain to help locate tumors that were not located in the recorded sites. Stimulating the right inferior parietal regions triggered a strong intention to move the contralateral hand, arm, or foot, whereas stimulating the left inferior parietal region produced an intention to make the movements of speaking. When stimulation strength was increased, subjects believed they had actually made such movements, even though monitoring of the relevant muscles showed no signs of muscle activation.

As with all such studies, the investigators only considered a subset of all the brain areas that are known to be involved in willed actions. For example, there were no electrical stimuli delivered to frontal cortex areas that are known to be involved in generation of intent. Just because realization of intent is generated out of the parietal cortex, that does not mean that intents was generated there. Even so, whenever intent is generated, it clearly must precede the realization of intent, and their studies clearly showed that realization of intent can occur without movement.

This result does not fit the zombian theory, for there was clear sign of willed action even when no movement occurred. This paper cites earlier work by Fried and colleagues who showed that low-intensity electrical stimulation of the supplemental motor cortex in humans caused an urge to move. Stronger stimulation caused actual movement.

The lay press has commonly claimed this is proof of free will. I don’t go that far, because the data just show that the parietal cortex enables people to be aware of their intent, not whether that intent was first generated consciously. There is also the problem that the really crucial point was not tested. Namely, can subjects distinguish between a stimulus-induced feeling of intent and an internally generated actual intent? Only if such distinctions cannot be made can one conclude that stimulus induced feelings are a valid index for testing free will.

I conclude that since parietal stimulation never caused movement, it may be that parietal cortex is a “reporter” region that generates realization of a free-will urge that is generated elsewhere in an area that provides input to pre-motor and motor cortex.

On the other hand, this work is a refreshing departure from Libet-type experiments. Because the focus is on stimulation, the limitations are of a different kind. One might object that the seven subjects involved had abnormal functions because of the nearby tumors. However, the consistency of effects suggests that the results might have been obtained in tumor-free subjects.

The authors noted the earlier research on the cognition of intention and the zombian theory. But they were careful not to endorse (or criticize) the zombian theory. Instead, they made the limited interpretation that the will to move precedes movements and even intended movements that do not occur.

The Soon study (Soon et al., 2008) has been followed up most recently with electrical recordings (Christophel & Haynes, 2009). Not surprisingly, electrical changes from multiple scalp electrode locations occurred several seconds before subjects indicated a conscious decision to move. These results were intended to not only confirm the earlier fMRI results, but also as proof that such antecedent activity reflects subconscious decision making. The interpretative flaw remains: Decision making is *assumed* to be unconscious, with consciousness only having a reporter function. The increased activity at the time of reporting intent is defined as irrelevant to making a decision, which presumably was made subconsciously. But where is the actual evidence? All such data really prove is what we already know: that there is antecedent neural activity.

## Twelve Interpretive Issues

I think that zombians commit at least 12 major fallacies of logic or accept insufficient data in interpreting experiments of this kind:

### 1. Increased neural activity has alternative interpretations that have not been ruled out

Haggard and Eimer (1999) reported that the potential has two phases, an initial stage where the readiness potential is evenly divided across the two hemispheres and a later lateralized phase. The lateralized phase actually coincides with conscious awareness and therefore could be a causal correlate of a freely willed action or at least guided the required movement. This finding raises the possibility that willed intentions may intermingle subconscious and conscious elements.

In the early ramp-up of the electrical signal, the change could signal that a movement command was about to be issued or that there was intention to move. Those are not identical processes. That intention could have been generated elsewhere, in areas of brain that were not being monitored. Maybe the processing of intention triggers the ramp up at the same time as the processes that were signaling the awareness of the intention.

Another problem is that increased neural activity in a given brain area may not be limited to just one function. While that may not be true in motor cortex, the SMA and certainly frontal and cingulate cortex perform more than one function. Do not circuits in these areas overlap with other cortical circuits that process other things? Could not these circuits be recruited into a larger network that generates free will?

Zombians assume that evolving brain activity prior to conscious awareness of an intention to act is associated only with preparation for movement. That activity could reflect other kinds of processing, and Trevena and Miller (2010) have recently tested this assumption. They compared electrophysiological activity before a decision to move with activity present before a decision not to move. There was no difference in the signal, and that argues against the conclusion that the increased neural activity reflects preparation to move. Now, we have to consider the possibility that this antecedent neural activity actually reflects conscious processing of the respective decision to move or not move. Trevena and Miller suggested that the neural activity change may “simply develop as a consequence of some ongoing attention to or involvement with a task requiring occasional spontaneous movements” (p. 454).

There was another significant difference in the methodology used in these two studies. The Libet paradigm tracks when subjects spontaneously intend to move, whereas in the Trevena and Miller study, subjects were given a tone cue, unpredictably presented, at which time they were to make a decision to move or not move. Gomes (2009) argues that the two conscious decision processes are therefore not comparable, though he concedes that the decision is spontaneous. He also disputes the claim that there was no difference in the observed antecedent electrical potential, noting apparent differences in time course and amplitude of the readiness potential. So this debate will continue to rage.

One fundamental aspect of free will is the decision to act or not act, which was the choice available in typical Libet-type experiments. An fMRI study of what the brain does during intentional inhibiting revealed that a fronto-medial cortical area was more strongly activated when people willed a manual action, but then willed to cancel it than when they completed the same action (Brass & Haggard, 2007). Thus, this study revealed an important area of cortex, not motor cortex, that is involved in controlling choice behavior. Moreover, this area appears selective for decisions to inhibit. This area is distinct from the areas that generate intentional actions, attend to intentions, or select between alternatives. The latter conscious decisions to act apparently arise elsewhere.

My take on this dispute is expressed in the title of this paper: Simple experiments are not so simple. Future studies should examine appropriate baseline measures of unspecific neuronal pre-decision activity in order to demonstrate the existence of decision-specific neuronal pre-decision activity.

### 2. Decisions are not instantaneous

One question that both neuroscientists and philosophers who endorse Libet-type experiments usually avoid is this: Why do we think that a decision is instantaneous? What we consciously think could well be spread out over time. The process can be on-going but our realization captures the process only as a snapshot in time that suffices to label the decision but not the process. A few philosophers, such as Daniel Dennett (2003), suggest that conscious decisions are smeared about in time and space (and thus correspond to distributed processing throughout many parts of the brain, not just the motor cortex neurons that control finger movement). Libet-type experiments seem to fail to accommodate the possibility that a freely willed intent can be generated early on, but consciously realized later because decisions have to “ramp up” until a threshold is reached when a person realizes the decision has been reached (Cleeremans, 2008).

Moreover, in experiments like this the subject continuously wills to perform the task and to do so within the rules of the experimental paradigm. The only thing at issue is when to act. Even the decision of when to act is not instantaneous. Even if not verbalized with silent self talk, the subject has to monitor time and think consciously about what is an appropriate time to act. “Has too much time elapsed since the last act? Should I use a set pace of responding or use a semi-random pattern? Do I know which response pattern I am using? How often do I change my decision to act now or defer it?”

In a more complex situation, decision-making is an on-going process. We weigh the evidence. We lean one way, then the other. Finally, the preponderance of evidence and the weights we assign to it lead to a decision. The decision itself may have been instantaneous but its process could have been dominated by free will choices spread out over days, months, or even years.

In Libet-type experiments, the neural activity begins its ramp up about the same time as the conscious urge to move occurs and reaches its peak at the time of actual movement. There is no necessity to believe, as many scientists do, that a movement has to be caused by an instantaneous burst of firing from one place in the brain. Causal activity may arise from many places within the brain that are functionally linked both sequentially and in parallel in ways that spread the process out in time. How can we know, for example, that the instant in time that Libet chose to observe crudely and perhaps inaccurately was the only or even the key instant at which the decision to move fingers was made? Where in the brain are such decisions made? Was that neural activity monitored? This same point is made by Roger Penrose (1994).

### 3. Conscious realization of intent is not instantaneous

Libet (1973) himself was the first to show that conscious realization itself can take at least 500 ms. In human subjects who were electrically stimulated in the somatosensory cortex, the stimulus had to be delivered for 500 ms or longer before they realized the sensation.

Given that there is nearly a half second delay in the appearance of a conscious threshold sensation, Libet and co-workers had to explain how persons report experiencing the sensation as if there is no delay, when the sensation is elicited by a stimulus pulse to the skin. They proposed that the time of the delayed awareness is subjectively referred backwards in time. This view has been criticized on the grounds of lack of evidence. However, this whole issue has been recently reviewed by Libet (2006), and he claims that he has proved the backward referral hypothesis. Stimulation of the subcortical cerebral pathway for specific projection to somatosensory cortex required the same 0.5 s repetitive train of stimuli to produce a threshold conscious sensation as did somatosensory cortex. However, unlike the cortex, each stimulus pulse in this subcortical pathway elicited a primary evoked response like that with a skin pulse. The very first stimulus pulse in in the thalamic medial lemniscus or VPL nucleus, it is claimed, provides the hypothesized timing signal for backward time referral. For technical reasons, Libet used stimuli at the threshold intensity for a 200 ms train of pulses effective for a conscious sensory experience.

In experiments of this type, two things have to be done at more or less the same time, neither of which can be assumed to be instantaneous. In addition to deciding when to move and realizing a willed decision has occurred, the subject also has to think consciously about the time indicator for the decision. To do this, one must be consciously aware of the indicator and integrate the movement into that awareness. Does the subject think about the clock in the context of “I am about to move and must make sure I note the time?” Or does the subject force a spontaneous movement and then switch attention, after significant delay, to note the time? Both the decision and the time recognition need external validation. Can the subject know for certain a decision has been made if he does not get visual or other confirmation of the act? How long does it take for proprioceptive or visual feedback to confirm the act has occurred and that the clock really showed X number of seconds?

Another line of evidence that it takes a while for a conscious realization to become manifest comes from the research of Grill-Spector and Kanwisher (2005). With images presented in sequence, for example, they found it takes up to about 100 ms to accomplish the correct conscious recognition of an event. In other words, subjects need this time after seeing an object to process in consciousness what it was and what category of objects it belongs to. At all time lags, accuracy was the same for detection that something was seen and its category, but was substantially less for realizing or identifying what the object was. On average, 65 ms were necessary for identification of what the object was than for its categorization, even when accuracy in the categorization and identification tasks was matched. Using visual images to test the time for conscious recognition of an event is especially useful evidence, because vision is an exceptionally high-speed process in the brain, very likely to be much faster than the conscious processes needed in a Libet type experiment where one must decide to move, determine what to do and with what body part to use to do it, and be consciously aware that these events have occurred. In other words, you can make a conscious decision to act, but it may take you several hundred milliseconds to become consciously aware of what you have decided.

To conclude this argument, the causes for a given decision or behavior are so numerous and interconnected that we can’t identify and understand them all. When it comes to consciousness, there is no good reason to expect to find any particular point in time when consciousness realization begins and ends.

### 4. Decision-making is not the only process going on

Pacherie (2009) points out there is more to causation than the initial triggering. The event or action, such as deciding to press a button, may be shaped by an ongoing set of mental processes. These may even overlap in time. Conscious intent could guide, if not trigger, part or all of these processes. Free will need not be the first triggering event.

Actually, there could be four conscious processes going on prior to movement commands in the Libet designed experiment. In such experiments, the subject could be thinking the equivalent of

1) “I know the rules of this game and agree to play by them.”

2) “I intend to move soon (and withhold movement in the mean while).”

3) “I realize and confirm that I have issued the order to move.”

4) “I notice and report the time I issued the order to move.”

Each of these is but one facet of consciousness, yet we need not necessarily witness all stages of a conscious choice being made. In some cases, we only witness (and monitor, as in the Libet experiment) the arrival of a portion of the conscious choice. Decision-making is a process, not solely an event. The same principle applies to subconscious decisions, but I make the point here because zombians seem to overlook the role of multi-step processes in conscious decision making.

Most theorists tend to ignore the full dimensions of these conscious processes, focusing only step three as the single important incident. They gloss over the role of steps one and two in biasing the relevant time-monitoring mechanisms and the movement systems involved not only in making the movement but also for reporting the moment of intent. Step 4 is usually not recognized to exist as a separate — and time delayed — process.

**Figure 3.**
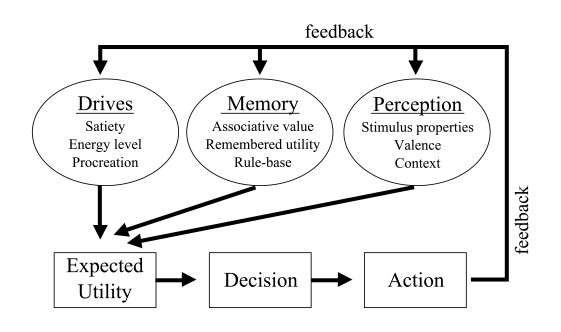
Constellation of processes that participate in making an intention, choice, or decision.

To help understand that complex cognitive processes cannot be explained by simplistic experiments, no matter how ingenious, let us recapitulate what could be happening during a decision to make a movement (Figure 3). External stimuli or even internally generated signals would generate a conscious decision to perform a given act. These signals could activate memory banks as a check on the appropriateness of the movement in the context of what has been learned about making such a movement. The reward system might be activated to assign value to the making of such a movement, weighing the expected immediate utility with the longer-term value. The emotional networks of the limbic system may be activated to see what level of passion, if any, is appropriate to the movement. Movement control networks have to be activated in order to plot a trajectory and to evaluate the correctness of the anticipated movement. There are pre-motor areas of cortex that are probably engaged in the planning for the movements that are to be executed. The single brain area monitored by Libet certainly should not be the temporal bench mark for deciding the time relations between conscious decision and engagement of motor control processes. A properly designed experiment would monitor other areas of the brain, preferably multiple areas at the same time, with monitoring protocols that could serve as a better indicator of when a conscious decision was made. Even free-will critic, Daniel Wegner (2002), concluded that multiple brain systems had to underlie the experience of will and that these areas do not seem to the same systems that cause the actual action.

### 5. Decision-making and decision-realization are likely to be separate processes

This could impose delays because both processes could require numerous synapses in widely distributed circuits, whereas the movement command can be executed via as few as two or three synapses of the pyramidal tract neurons in the motor cortex that project uninterruptedly to a lower-motor neuron in the spinal cord.

The earlier mentioned studies on the relatively long time it takes to realize a decision supports the notion of separate mechanisms. Recall also the Soon et al.’s (2008) demonstration of a full 10 s of activity prior to willed actions.

It is also possible that conscious realization processes are not complete until they are confirmed by feedback from seeing and feeling that the movement has actually occurred. Realization captures the *process* as a snapshot in time, but the antecedent process of realization could go unrecorded.

Finally, willing a finger movement is too simple to have much bearing on such conscious processes as the decisions made through introspection, planning a course of action that spans past and future, or analysis of complex events.

### 6. Not all intentions are for simple movements

Willing a stereotyped, well-rehearsed finger movement is too simple to have much bearing on such conscious processes as the decisions made through planning a course of action that spans past and future, or analysis of complex events. Why, therefore, would anybody be surprised at absence of a robust antecedent indicator of willed finger movement?

There is also the issue of the kinds of movement we wish to correlate with conscious intent. In speech movements, for example, we have all experienced high-speed conversation, clearly controlled by conscious intent to express thoughts, both spontaneous and in response to what is said by others. Consider all the thoughts one has to hold in conscious working memory to conduct intelligent conversation. We think consciously about what is in working memory as we use it. Libet-type experiments don’t seem to fit into such real-world conscious experiences. True, conversation often contains knee-jerk responses, no doubt subconsciously driven. But it is hard to defend a position that conscious mind is just an observer of a lively, intelligent conversation.

Finally, what are we to make of choices or decisions where no immediate motor act is involved? Recall the studies of Desmurget et al. (2009). What experiment could cast doubt on the free will involved in self talk, setting goals, making plans, adjusting attitude, developing belief systems, or any decisions or choice not involving action or active refusal to act?

### 7. Not all willed intentions are formed in acts of decision

Especially in the case of habits, decisions may have been made long before the initiation of an act. That is to say, as Mele (2009) points out, an intention to do something can arise without being actively formed from a decision process. Not only are some habits originally formed consciously, but the choice to deploy a habit may be made consciously and certainly, as Libet suggested, be vetoed consciously. Of course, once habits are initiated, they may be executed with little or no conscious involvement (Hommel, 2000). In the early days of human-movement science, Fitts and Posner (1967) formalized the commonly accepted notion that learning movement skills progresses from an early stage where consciousness directs the process, but in later stages, the movements become automated. Pressing a button is a skill so simple and so readily learned it becomes automated easily and quickly. Why would anybody be surprised that a button press could be done without being consciously driven? On what logical grounds can zombians leap to the conclusion that all behavior is automated? 

Additionally, conscious performance of a behavioral act can be of different types. In a typical free-will experiment, the subjects’ mental processes dynamically fuse the three categories of conscious intent: future, present, and motor (Pacherie, 2009). Present and motor intentions occur simultaneously with the behaviors they are guiding, but this is not true of future intentions. Further, a behavioral act can have three conscious components: know *that* we are doing something, knowing *what* we are doing, and knowing *how* we are doing it. I would add, *why* we are doing a given thing. All of these elements (future, present, and motor; that, what, how, and why) are present and confounded in Libet-type experiments and most others.

Even though neural mechanisms that cause these components of an act may not be accessible to consciousness, this is not proof there is no role of conscious intent. Intentions for any or all of these components might have an element of conscious causation. Moreover, all these components can be smeared across time, making highly suspect the introspective judgments about associated willed action and time.

### 8. Conscious decisions can be temporally uncoupled from the action

I may decide this morning, for example, to be more thoughtful toward my spouse. Opportunity to do that may not arise for hours, as for example, when I come home from work that evening. When the opportunity arises that evening to be thoughtful, do I have to re-make the decision? No, it had already been made hours ago. So, when I do nice things that evening, the new behavior resulted from a choice action at that moment, not a decision made hours ago. One could argue (but not test) that the evening’s behavior was generated subconsciously, but it could not have been driven by the process of making a conscious decision, because that had already been done.

Another example of uncoupling comes from studies of Galdi et al. (Galdi, Arcuri, & Gawronski, 2008). They tested the predictive effect of automatic mental associations of undecided individuals (as in deciding who to vote for in an election). The results indicated that future choices of undecided voters could be predicted by their current automatic mental associations, even when voters insist that they are undecided. Sometimes decided individuals had already made up their minds, even though they consciously insisted they were still undecided.

However, all such observations prove is that self-reported conscious decisions can be biased by subconscious influences. No surprise there. Once in the voting booth, the act of where on the ballot to check could still be a conscious choice where one has the option to endorse their bias or reverse it.

### 9. Introspection is an unreliable indicator of when a freely willed action is made

Introspective judgments about conscious intent are not necessarily reliable. In one study of this point, participants made conscious choices of presented face pairs, based on attractiveness of the faces. After a short delay, subjects were then shown their choice. However, experimenters covertly manipulated the relationship between choice and outcome as experienced by the subjects, yet subjects often failed to notice conspicuous mismatches between their intended choice and the outcome they were presented. Nevertheless, their introspective reports reflected a blindness to what actually happened. Subjects actually developed confabulations to account for the mismatches (Johansson, Hall, Sikström, & Olssonet, 2005).

Unreliability of introspection was found with an experimental design intended to objectify introspective judgments of awareness of intention by Kühn and Brass (2007).They used a stop-signal paradigm and an intentional-signal paradigm and found evidence they argued supported the zombian hypothesis. Specifically, subjects sometimes (note, not always) indicated free choices when reaction times suggested that they failed to stop the action. In a second experiment, misattribution of awareness of intention varied with intentional involvement during planning the action.

Moreover, introspection is not the only way to study free-will issues. Social psychology literature is often not considered in debates about free will, yet that field uses other than Libet-type tests to address the role of consciousness in decision making. With new experimental designs social psychology approaches might assist in distinguishing subconscious and freely willed actions.

One approach is the study of habits. Habits provide the utility of performing acts without conscious awareness, thus “making room” for doing things that necessarily require conscious processing. When habits are established, the very activation of the goal to act automatically enables the habitual response. When behavior is habitual, behavioral responses are activated automatically. However, results of three experiments indicated that the automaticity in habits is conditional on the pre-existing presence of an active goal which might be freely willed (Aarts & Dijksterhuis, 2000). There should be experimental designs that test the role of free will in both forming and breaking of habits.

It is argued that this sequential relation of goal and habit execution increases the likelihood that individuals rely on subjective experience particularly under conditions that prevent considering retrieved contents. However, this view is not supported by all experimental designs (see Kuhnen, 2010).

Another approach is that by Pessiglione et al. (2007), who used a paradigm that varied monetary rewards for which subjects exerted physical effort. Even when subjects could not report how much money was at stake, they nevertheless deployed more physical force for higher amounts. Thus, behavior was energized subconsciously by expected rewards.

A test of free will might be designed in which one reports knowledge of the contingencies in a situation in which a decision or choice was made. If such knowledge is recognized, the act is likely free willed. When contingencies are not recognized, subconscious processes caused it.

Alternatively, tests of free will might involve choices or decisions that are habitual (and therefore subconscious) with those that are first-time events (and therefore could be reasoned and freely willed). Of special interest in such studies would be electrographic indices that might differentiate habitual versus first-time choice events (see later proposals on EEG recording).

### 10. Inappropriate reliance on awareness of actions and time estimation accuracy

Conscious awareness of time is central to the issue of when decisions and actions are consciously or subconsciously generated. In self-reported awareness of a conscious decision, the issue is whether the intention occurred prior to action or if the awareness was reconstructed after the action occurred.

It only takes mention of a few studies to make the case that humans are not precise in their awareness of time compared with actual time on a fraction of a second scale. Ono and Kawahara (2005), for example, showed that subjects made major errors in time estimation when instructed to keep visual displays on a screen for a fixed time. Moreover, the accuracy was affected by prior priming experience with the images.

Ulrich, Nitschke, and Rammsayer (2006) review a variety of reports show that time estimation accuracy is affected by experimental conditions, such as stimulus modality, degree of attentiveness to time, and level of arousal. Their own experiments showed that time estimates were affected by prior expectations about visual stimuli.

One recent study (Moore, Wegner, & Haggard, 2009) examined the time accuracy that subjects had for the interval between their key press movement and a tone. The movement was produced either voluntarily or passively by a motor. Subjects grossly underestimated time intervals for both voluntary and involuntary movements. Three of the 14 subjects were so erratic in time estimation that their data were omitted from analysis. To manipulate the sense of agency, experiments included priming the subjects with thoughts relevant to the movement just before it was made. Timing estimates were modulated by such priming, becoming greatest for involuntary movements. A second experiment showed that this modulation depended on prime–movement (temporal) contiguity.

The key point of these findings, in the view of Synofzik et al. (Synofzik, Vosgerau, & Lindner, 2009), is that optimal cue integration seems to be the key to a robust sense of agency. This means, of course, that test designs may fail to affirm free-will intentions simply because cue integration was not optimal. In any case, the study shows unequivocally the unreliability of time estimation.

In the Libet experiments, the subjects could have been wrong in their reading of the clock. There could have been a lag of a fraction of a second between the time they made a conscious decision to move and the time that they noted and their brains packaged that information for verbal delivery to the investigators. Libet himself noted an error of about 200 ms in the subjects’ recall of the times when they first became aware of sensations.

Stanley Klein (2002) re-plotted Libet’s original data and found that observers had great uncertainty about the relative timing of events. He also points out that the Libet design required responses that were difficult to judge.

Several experiments document that it takes time to process visual information consciously. In an experiment originated by Nijhawan, subjects assess the timing of an object passing a flashbulb. The timing is exact: The bulb flashes precisely as the object passes. But subjects perceive that the object has moved past the bulb before it flashes (Nijhawan & Kirschfeld, 2003).

This suggests that the brain projects a moving event a split second into the future, seemingly working on old information. Apparently, the brain needs time to consciously register what the eye sees. In the context of a Libet type experiment, realizing the location of a clock hand could occur later than what the time actually was.

Various investigators have raised questions about the accuracy of time awareness under conditions specifically relevant to Libet-type experiments. For example, Joordens, van Duin, and Spalek (2002) directly examined potential biases in this task by asking subjects to make subjective timing decisions about a stimulus. Subjects consistently tended to report events as happening about 70 ms later than they had actually occurred.

A specific re-examination of time awareness accuracy in the Libet paradigm has been reported by Danquah, Farell, and O’Boyle (2008). Using the control condition of the Libet method, subjects had to judge the time of occurrence of a stimulus relative to a clock indicator of time. Response accuracy varied systematically with the sensory modality of the stimulus and with the speed of the clock. If these indicators of externally observable events are inaccurate, the researchers suggest that their time estimation may also be inaccurate for endogenous events.

In addition to reaction-time lags and errors, there is no accurate coupling of perceptual awareness of time and actual time. Many scientists are now starting to study how the brain is aware of time and tracks it in relation to events. Although these studies are not done in the free-will context, they are very relevant because they teach us about the brain’s limitations in being aware of time and events in time.

Awareness of time is only one indicator of how well humans are aware of their actions, and it can be argued that humans have awareness limitations that go beyond time awareness. For example, a just published paper reports that awareness of our actions depends on a combination of factors involving what we intend to do and what we actually did. Sarrazin, Cleeremans, and Haggard (2008) report an experiment in which subjects were instructed to reach consciously for a target that jumped unpredictably on some trials. Subjects were to express their expectation of a target shift, point at the target as fast as possible, and reproduce the spatial path of the movement they had just made. The last step of reproducing the trajectory was taken as an index of the awareness of the previous action.

The accuracy of reproducing the trajectory was measured in terms of the degree of movement undershoot or overshoot. On trials where subjects thought there would be a target shift, the overshoot was greater and the undershoot less than on trials with lower expectancy. Thus, conscious expectancy affected the awareness of what had taken place.

Time-awareness accuracy is confounded by the likelihood that the whole process of decision making and monitoring has many elements that combine subconscious and conscious processes. Of all these processes, Libet only observed that the “action” stage had only started before subjects thought they had issued a command to move. The Canadian scholar, Merlin McDonald (2001) makes my point by stressing that the time scale used in Libet-like studies is too short to adequately capture all conscious processes. In the Libet study, the actual movement did not occur until after subjects thought they had decided to move, which allows for the possibility that the processes above could have participated in a conscious will to move. Some portion of these processes occurs at a subconscious level that could have primed the motor cortex to start a readiness ramp up of activity to await final confirmation from conscious decision making.

And how do we explain other kinds of decisions that are so rapid that long preparation periods are not possible? For example, one news story on free-will research began this way: “You might think you just decided to read this story on a passing whim — but your brain actually decided to do it up to 10 s ago, a new study claims.”

The problem here is that I made that decision to read in a split second, because I had just clicked a hyperlink to take me to the Web page where the story was posted. My brain could not have made a decision much in advance, because my brain did not know such a site existed more than a few milliseconds earlier. It is still possible, of course, this rapid decision-making occurred in my subconscious before I realized I made it. But in my conscious mind, I certainly considered whether following a hyperlink was likely to be worth my time, and I could have rejected whatever decision was fed to my consciousness from subconscious processes.

### 11. Unwarranted extrapolation to all mental life

Just because subconscious choices are made prior to conscious awareness in one task is not proof that all mental life is governed this way. How can intelligent people extrapolate and generalize a simple movement to all other conscious processes the brain performs? How can this kind of methodology possibly be appropriate to test for free will in such conscious cognition choices as deciding on an optimal plan, a correct problem solution, what to conclude, the appropriate interaction with others, which words to use in conversation, or what attitudes and emotions to embrace?

Complex tasks are probably performed in different ways than simple ones. Yet zombians seem to assume that the mechanisms in this simple button-press task are the same as in such complex tasks as conversation, planning, attitude adjustment, introspection, problem analysis, etc. It may be that the reflex-like button press response is so simple that the unconscious mind performs it and has no need to assign or recruit assistance from conscious mind in making the decision.

**Figure 4.**
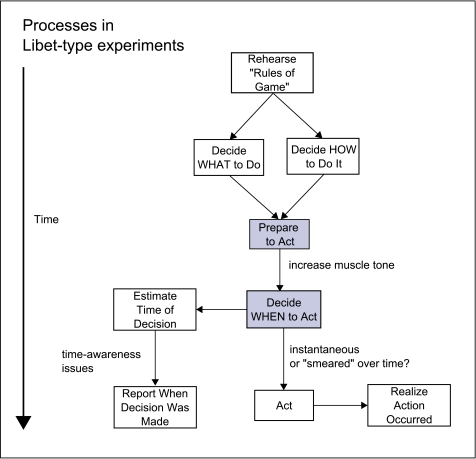
In a typical Libet-type experiment, it seems possible that all of the processes, except for the two with shadowed backgrounds, are performed consciously. Note that they are intermixed in time and they cannot be interpreted unambiguously.

All of the experiments used to support the zombian conclusion are of the same basic and quite limited type. But there are different forms of intentions and any given form may not be as simple as it seems. Many neural processes are going on that are not taken into account, even in the simplest designs (Figure 4).

This scheme more correctly describes, I think, what the brain must be doing to make the simple finger movements in the Libet-type experiment. This scheme should make clear why the measurements in such experiments cannot possibly be an accurate reflection of all that is going on. More specifically, there is no way to show that the ramp up in motor cortex activity occurred before a long sequence of operations involving intent generation, conscious working memory of the “rules of the game,” the instant of intent realization, the realization of the time of intent, and the linguistic preparation for declaring the information. Some of these processes, such as ongoing working memory of the “rules of the game,” are clearly present before ramp up of motor cortex activity.

A series of processes occur in parallel over time. Rehearsal of the “rules of the game” occurs continually. This is the context in which everything else occurs. One process involves first the decision to make a movement at some point. This is followed by consciously informing oneself that now is the time for a movement to be made (“what to do”) and also to choose the correct hand to activate the actual motion (“body part to use”). Then, after significant delay, the conscious mind realizes that these decisions are now complete and readies itself for action. This is followed by the activation of motor cortex to prepare for and execute the movement. The brain has to decide to split or divert attention from the movement commands to noting the time. Time of decision has to be estimated and consciously realized for subsequent reporting.

In parallel, a set of processes is triggered, first involving integration of the command to move and to do so with the right hand. This is followed by the activation of motor cortex to prepare for movement and finally initiate the movement.

The most salient point is that many of these cognitive processes have to be held in conscious working memory, in order to perform the expected task. These working-memory tasks are smeared out across time and there may not be any single electrophysiological signature of their occurrence

**Figure 5.**
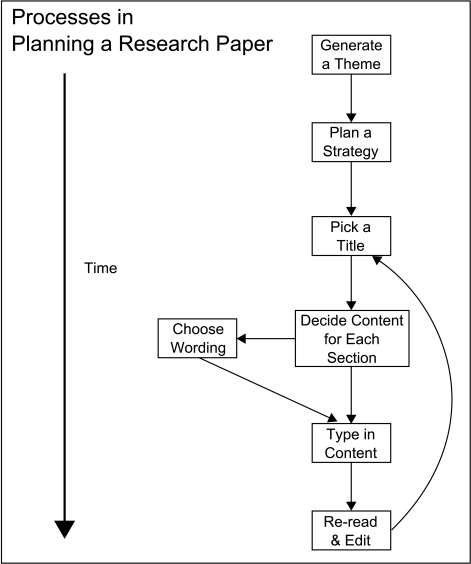
Simplified outline of the stream of conscious decisions needed to write a scientific paper. The research reviewed here provides no support that such operations are all performed subconsciously and that conscious mind has no role in the multitude of decisions, many of which overlap in time.

Next, compare this kind of processing with what happens in many areas of cognition. For example, consider the process for writing a scientific paper, assuming all data have already been analyzed (Figure 5). Even though it is unclear to what extent subconscious processes operate, it is clear that conscious thought dominates all of these steps on a continuing basis. How relevant can Libet-type experiments be?

### 12. Conflicting data or interpretations are ignored

Recall the data of Soon’s group (Soon et al., 2008), which showed increased activity in two regions of the frontal and parietal cortex a full 7-10 s before conscious awareness. This was considered evidence of unconscious motor preparation. There is no basis for believing it takes 10 s for unconscious mind to prepare motor pathways for a button-press movement. Why do zombians assume this predictive change reflects motor preparation instead of the processing of free will and other cognitive functions associated with the “rules of the game?” These areas of brain normally have conscious functions and not movement functions. Is this not bias?

Zombian bias may even keep investigators from looking for evidence crucial to the argument; namely, neural representation of intention. Yet, there is enough evidence to indicate there are neural representations of intention, as for example in the Desmurget study (Desmurget et al., 2009). A slow time scale allows for conscious awareness of intent, development of plans and “on-the-fly” adjustments. Consciousness allows us to think in the future, to anticipate what we need to do to get what we want and to plan accordingly. Such intentional planning has a neural representation and can even be detected experimentally in animals. In one such study, Sam Musallam, Richard Andersen, and colleagues (Musallam, Cornell, Greger, Scherberger, & Andersen, 2004) eavesdropped on neurons in a planning area of monkey brain. They put electrodes in an area of cortex that was known to be required for planning, but not actually making, arm movements to reach a target. The planning area in monkeys is a small patch of cortex just above the ears. Monkeys were trained to “think about” a cue presented on a computer screen that told them to plan a movement toward an icon on a screen that had just flashed on a screen in one of up to eight locations. Each location was associated with a certain firing pattern in the planning neurons. Here is a clear case where the will to do something was established long before any action occurred. While monkeys thought about the required movement, computer analysis of the firing patterns of these neurons could predict what the monkey was intending to do — tantamount to reading the monkey’s mind. The researchers knew that it was intention that was represented, not actual movement or even planning for movement, because the monkeys were trained to get reward only when they withheld actual movement but nonetheless made the correct planning, as indicated by their neural firing patterns. Whether or not the monkeys were consciously aware of what was going on is another question. But it is clear that these animals have a mind that contains neural representations for decision processes, and these neurons are active prior to planning for motion or even in the absence of movement.

If a monkey can make decisions for the future, surely we can. Of course, planning can be subconscious or conscious, and this argument is moot, if one believes that monkeys are incapable of consciousness.

## Common-experience Examples of Free Will

Numerous common-sense examples could be constructed to illustrate complex situations wherein conscious intent can occur. The examples I give are all based on presumed conscious free will to make certain movements. This limited view is chosen because the research that suggests free will to be an illusion has been based on intent to make the most simple kinds of movements, such as a button press.

Here is one example: You are driving a car in heavy traffic and another car runs a red light, pulling into your path. You can realize the full nature of the emergency and intend to turn the steering wheel appropriately and move your foot off of the accelerator and onto the brake pedal long before you can make such movements. You may not be able to avoid the accident that you consciously intended to avoid. The analysis of the emergency, the intent to make certain movements, and the motor execution is all completed in a fraction of a second. And we need to take into account the fact that a decision can be made but not consciously detected for up to a half second. How likely then is it that all this was figured out subconsciously, then conscious awareness was engaged, and then conscious awareness was realized in that same instant? How can the responses be generated subconsciously when the subconscious has not been preprogrammed for such movements? From beginning to end of the episode, conscious intent processes are clearly operative. Though zombians reject such analysis, can they falsify the hypothesis of conscious intent?

Here is another example that football fans can relate to: In almost every game there is at least one play where a pass receiver drops the ball because he was consciously thinking not only about catching the ball but also about defensive backs that he heard thundering toward him and was thinking about the moves he would make after the catch. All this was going on in conscious mind long before the brain issued the movement commands needed to catch the ball. You might argue that the preparation to move was triggered before all the conscious realizations about the pass-receiving context, but that can’t be measured. As in the car accident case above, there is no way the subconscious is preprogrammed to make all the right movements, given all the variables involved and the uniqueness of every pass-catching challenge. In any case, it seems clear that conscious thought and decisions were being made well before complex motor commands were issued and adjusted in the last few milliseconds to adjust to the ball’s trajectory and speed to accomplish the desired movements.

True, intent to move might be preceded by unconscious preparations and rudimentary alternative sets of muscle commands that could be considered for movement. But it is hard to argue that conscious thought about how and when to move is preceded solely by unconscious processes. Conscious planning, by common-sense definition at least, commonly precedes action. Scientists will point out that common-sense can be wrong. But so can scientific dogma.

If subconscious mind does everything, and conscious mind is merely a by-stander that may intervene on occasion, we have a problem in explaining the decisions and conclusions we make in:

1) Attitudes and beliefs we choose to make as a result of introspection.

2) Conclusions we choose to make from literature, poetry, art, or music.

3) Deciding what words to use in rapid conversation.

4) Choices we make about time (past, present, and future).

5) Intentions we use in early-stage learning, such as riding a bicycle or touch typing.

6) Deciding what to believe in politics, religion, etc.

7) Decisions to take or avoid responsibility.

8) Choices that emanate from conscious analysis.

9) Choices made in developing plans for the future.

10) Feedback adjustments to ideas, attitudes, emotions, and behavior?

The subconscious mind surely participates in all of these human cognitive activities, but to presume that all of these activities are governed *only* by subconscious mind is an assault on human reason. Only a few scientific studies of free will have been performed, and each has involved only decisions to make simple movements that one already knows how to do. These studies have seriously flawed assumptions and interpretations. Also, each of these studies is contaminated by the requirement of pre-requisite processing needed to hold in conscious working memory the rules of the experimental game. In other words, I think that scientists who argue against free will have jumped to conclusions — hardly a judicious scientific stance. Until science provides *evidence* (as opposed to speculation cloaked in pseudo-scientific garb) it is scientifically irresponsible and dogmatic to insist there is no such thing as free will. It seems to me that such scientists are left with arguing from authority, as indicated by their citing Darwin and Einstein as zombian allies (Sommers, 2007).

Cognitive tasks come in wide variety, and a “one size fits all” explanation about whether or not they are zombian is not appropriate. Certain musculoskeletal actions require consciousness, not only for monitoring the action but in some cases for initiating it. Not all elements of a consciously initiated action are freely willed; in fact most elements may be controlled subconsciously. Perhaps button pressing falls into this category.

There are several forms of intentions, according to Pacherie (2009). These include intentions to do something now or do it in the future. There are also specific motor intentions, commonly the focus in free-will experiments. Motor intentions occur in two classes, control and guidance, and they can occur on a time scale of a “micropresent,” which only partially overlaps the present conscious state. Pacherie argues that conscious intentions can cause a behavior without necessarily giving rise to an experience of conscious will. If so, lack of evidence for free will is not evidence for zombianism.

The differences between conscious and unconscious actions are subtle (Morsella, 2005). No less subtle are the differences between conscious actions that are freely willed and those that are not. Consciousness can simply exist as a state (“I think, therefore I am“), or it can be a free-will agent. Of course both elements can occur concurrently.

Morsella (2005) likes to focus on task demands and whether or not they are “penetrable“ to conscious operation. The difference between conscious and unconscious processes, he says, lies in the kinds of information that have to be taken into account in order to produce adaptive behavior. Motor acts in the typical free-will experiment are so simple they may not even need to be penetrable by consciousness. One could employ similar logic to assert that some conscious intentions are so simple they don‘t require a free-will trigger, but others so complex that they could not occur without it. More complex motor acts may require, for example, planning, which perhaps cannot be completed without some element of freely willed choices and decisions. In a Libet-type experiment, the motor act may be so simple that it can be performed with minimal free-will intent, which, lacking robustness, is poorly and slowly identified through introspection.

Morsella (2005) does seem to suggest that most of consciously initiated action is not freely willed. His supramodular interaction theory envisions choices and decisions to arise from dynamic interactions of multiple response systems. That is, these systems respond to contingencies to generate intent and motor command. In that sense, the over-all phenomena could be conscious and even willed, but not freely willed. This view, of course, raises the thorny issue of what is a free choice. Obviously, all choices can be influenced by prior learning. But what about choices for which there has been no relevant past expe-rience? When such choices are made consciously, they could satisfy a free-will criterion if the task were sufficiently novel and complicated. In high-speed conversation, for example, consciousness may be an essential mechanism for solving the problem of integrating processes in a largely parallel brain that must satisfy the demands of a skeletal-motor system (lips and tongue in this case) that can express intentions and corresponding actions only one at a time. In other words, you couldn‘t perform the speaking task without instantiating free will.

Zombians reject common-sense arguments. Yet, I have not seen anyone make the following point, which I believe to be irrefutable: In learning a new skill, such as playing the piano, there is no way the subconscious mind can control movements in the beginning, because it has no way of knowing what to do. Only the conscious mind can choose which keys to press because only it knows what should be done. If that is not free will, what is?

## Personal Responsibility

The free-will issue is more than an arcane scholarly argument. There are serious adverse social consequences to the zombian view. Positions become politicized. In a zombian world, people are more likely to be victims and less able to change maladaptive attitudes and behaviors. Thus, society and government must help them do what they cannot do for themselves. In a free-will world, people can choose to extricate themselves from misfortune.

This is not to advocate teleologically that free will exists because it is personally and socially adaptive. On the other hand, is it possible that free-will has great adaptive value and therefore evolved through natural selection forces? Evolutionary considerations should not be dismissed out of hand. Darwin could have made this point, but chose to reach an opposite conclusion.

If we have no free will, then there is not much we can do to improve ourselves or our plight in life. Or even if there are things that can be done to change us and our situations, the approach will surely have to be different if we can’t initiate the change by force of our free will. The government or schools or some other outside force must program our subconscious.

The argument is central to the issue of personal responsibility. To believe in the absence of free will creates an intolerable social nihilism. If there is no “I“ in charge, then there is no reason to demand or expect personal responsibility. All manner of bad brains and bad behavior can be excused. If we believe there is no free will, how can we defend our criminal justice system? If people cannot make choices freely, and if all their decisions emanate from subconscious processes, then how can we hold them responsible for unacceptable morals or behavior? Criminals should only be given punishment that reduces the likelihood of preventing further crime. The brain committed the crime. If we have no free will, it is inhumane to punish criminals or even terrorists. Indeed, the only justification for locking anybody up for misdeeds would be to protect society from further crime. Capital punishment has to be banned, as indeed it is in many parts of the world. In the minds of some, criminals are victims. It is true that exercise of personal responsibility is harder for some than others. To be sure, most murderers have been found to have a standard profile that includes childhood abuse, and some kind of neurological or psychiatric disorder (Gazzaniga, 1998). But many non-murderers have a similar profile. How can lack of free will explain such difference? The monstrous magnitude of zombian nihilism requires us to reject cavalier acceptance of research that purports to show that there is no free will.

The reality is that most people have brains that can learn social norms and choose socially appropriate behavior. Ignoring those norms is a choice. How can anyone seriously contend that people have no conscious preferences, that we are driven only by impulses and desires? How can anyone contend that all our impressions, beliefs, value systems, and preferences are not molded by conscious choice? How can anyone seriously argue a person is not responsible for criminal and evil behavior?

Responsibility is not only a social construct, it is also learned by the brain. And the brain has the power to make learning choices that are not easy. A terrible childhood, for example, need not condemn one to an immoral or underachieving life. Conspicuous examples of willful rising above their environment include Abraham Lincoln and Thomas Huxley. Sigmund Freud was a cocaine addict. George Patton hallucinated. Merriweather Lewis, of the Lewis and Clark expedition, was a manic depressive.

Also, some brain abnormalities are created by the lifestyle and thought and behavioral choices that a person freely chooses. You will probably mess up your brain by snorting cocaine or smoking pot, but that behavior is something you chose to do. You may program your brain badly by associating with the wrong people, but again, that is a choice not a necessity.

**Figure 6.**
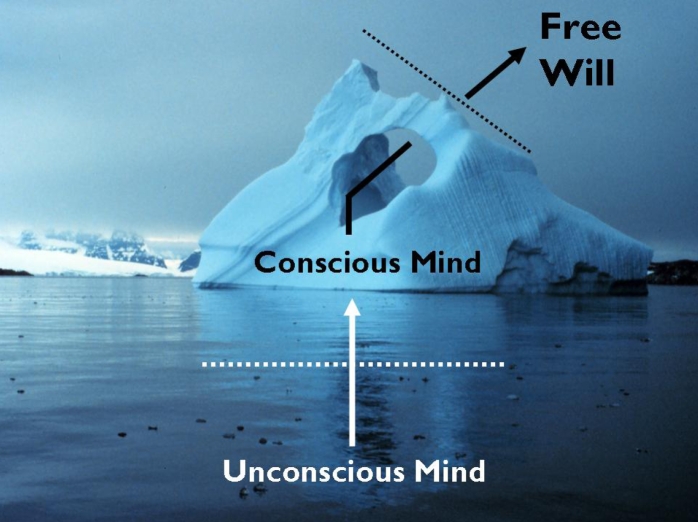
Emergence of free will from brain operations – a traditional view. Unconscious mind, originating in the spinal cord and brainstem, forms a substrate for developing a subconscious mind (white arrows and dotted line), which in turn can yield a conscious mind from which free will can emanate. Note that conscious mind is shown as the “tip of an ice berg,” beneath which lie more basic neural processes.

The desire to do something can arise from subconscious compulsions. But it can also reflect operation of the conscious mind. We can will ourselves into thoughts and actions. A way to think of the relationship of mind to free will is illustrated in Figure 6.

## Proposal for Next Generation of Experiments

If free will exists, then there should be some neural correlates when such will is being exercised. No one knows what those correlates are, mainly because they haven’t been looked for. Primitive assumptions about neural mechanisms of consciousness underlie many of the limitations of free-will research. Electrical recordings, brain scans, or stimulation of any one area of brain cannot provide much information about consciousness. Consciousness is not a thing in a place, but rather a process in a population, and that population undoubtedly is engaged in widely distributed parallel processes of complex-system dynamics. The current technology best suited for study of consciousness is the EEG, especially when quantified in terms of frequencies and coherence relationships among various brain areas at successive points in time.

Free-will research frequently has put the “cart before the horse” with attempts to use neural activity indicators of intent, choice, or decision-making, when we do not yet understand the neural activity that causes consciousness, much less any free-will consciousness. Study of the topography of oscillatory synchronization currently holds the most promise for identifying neural activity that causes consciousness. Once that is accomplished, we should be in a better position to use those objective measures to identify what is free will and what is not.

Almost certainly, free will emerges from a distributed process in neocortex, which provides the substrate for consciousness itself. One might monitor multiple neuronal activities within appropriate cortical columns. For example, if the willed task involves vision, multiple columns in visual cortex should be monitored. Perhaps changes in impulse onset/offset, firing rate, change in firing rate, or sequential interval patterns will be seen in certain neurons. Perhaps there will be changes in oscillatory frequencies of field potentials or in coherences with oscillations elsewhere or with other frequencies.

I suggest that there might be a global electrical marker for conscious decision making: synchronization of brain-wave oscillations at multiple locations. Degree of synchronization can be frequency specific, involving shifts in coherence among various brain areas and even among oscillators of different frequency. In my laboratory, we noticed that when subjects made a conscious decision about which mental images were present in an ambiguous figure, there was significantly increased synchronization in specific frequency bands across widely distributed scalp locations (Klemm, Li, & Hernandez, 2000).

Note the advantage of the ambiguous-figure paradigm. The physical stimulus on the retina can remain the same, while one alternative image is held in conscious awareness and at the same time the alternative image is held subconsciously. Moreover, an experienced subject can choose which image to hold consciously and which to suppress. Subjects can also control how long they hold a given percept. We used wavelet analysis, which allows one to track frequency changes as a function of short epochs of time, which is not feasible with conventional spectral analysis. We also found, much to our surprise, that synchronization occurred in multiple frequency bands, a finding that has also been reported by others (Makeig, Jung, & Sejnowski, 1998). For example, in a study of selective visual attention, multiple coherent EEG oscillatory components were observed to be differentially modulated by specific conscious events. It is also possible that a marker for conscious will action is the sudden synchronization of two or more oscillation frequencies with each other.

Whenever a person switches percept in an ambiguous figure stimulus from one alternative to the other, some aspect of cortical signals synchronizes. The obvious interpretation is that this is a correlate of conscious perception. But it is also a correlate of decision making; that is, we decide whether we are seeing a vase or a face. Subjects can, through force of will, choose which percept to hold in working memory. In fact, for many such images, many subjects have to extend considerable mental effort to perceive one alternative image because their default percept is so strong. Since oscillatory synchronization is so tightly associated with this process, this may be the clue that free will is enabled by synchronization of certain oscillations. An experiment could readily check for changes in coherence patterns when one freely wills to hold the difficult percept in consciousness as compared with patterns during the default percept. This does not prove there is no preceding subconscious EEG correlate, but the experiment might benefit from including a time indicator, of the Libet or Soon type, for when subjects realized they wanted to force perception of the difficult alternative image. If synchronization changes indicative of intent occur before the indication of conscious intent, it might support the zombian hypothesis. However, we would still face many of the faulty assumptions mentioned earlier (intent processes are smeared in time, extra time is needed for realization of intent vs. generation of intent, etc.).

A step in the right experimental direction is the experiment reported by Daeyeol Lee (2004) at the University of Rochester. He monitored the level of coherent oscillations in electrical activity in the supplemental cortex of monkeys in a task in which they made a predictable series of hand movements as they integrated sensory signals with expected reward. Movement performance was influenced by both the position of movement and the location of the rewarded target, but only the expected reward affected the degree of synchronization. I don’t claim that monkeys perceive these things consciously, but coherence of neuronal activity clearly seems to be a marker of something different from the amount of activity.

Another useful illustration of the value of EEG synchronization is found in the work of Melloni et al. (2007). The neural signals that differentiate unconscious and conscious thinking might be found in oscillatory synchrony of brain field potentials. In one test of such a possibility, subjects were evaluated while processing visible and invisible words in a delayed matching to sample task. Both perceived and nonperceived words caused a similar increase of local gamma oscillations in the EEG, but only perceived words were associated with transient synchronization of gamma oscillations across widely separated regions of the brain (Melloni et al., 2007). This parallels our own observation that sudden synchronization appeared at the instant when subjects viewing an ambiguous figure suddenly perceived the alternative image that had been previously inaccessible to consciousness.

Physiological correlates of presumed free will might benefit from testing under multiple-choice conditions. This overcomes many of the simplistic assumptions in dichotomous two-step designs that compares a free-will possibility with a no-free will baseline. It also would allow an experimenter to manipulate comparative strengths of choice options.

To summarize, I think this critique shows enough weaknesses in the zombian theory to warrant a new generation of experiments aimed at testing the possibility that there is neural representation of free will.

**Figure 7.**
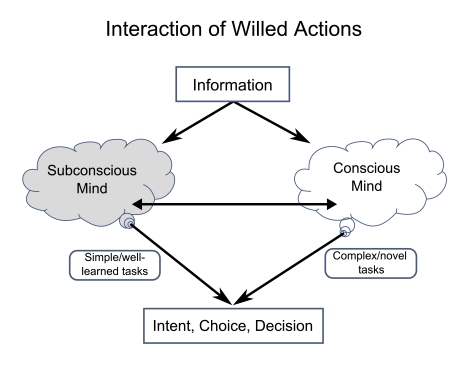
Embodied brain deals with external and internal information through joint action of subconscious and conscious operations, with the preponderant influence determined by the nature of the task. Simple, well learned, or habitual tasks may not require much freely willed influence. However, complex or novel tasks may not be possible without conscious “free-will” guidance.

## Concluding Philosophical Perspective

Commonly, we think of neural events as causing bodily movement as well as consciousness, and assume from zombian research that consciousness cannot cause neural events. This view treats consciousness as some kind of ethereal, out-of-brain, non-physical entity. But suppose that consciousness itself *is* a neural event! In that case, conscious intent would have a physical reality in the brain and would of course be able to influence other neural activity. Our current inability to describe consciousness in neurophysiological terms does not mean that this possibility is not accessible. In another paper, I attempt to describe a new way to think about and test conscious functions in neurophysiological terms.

Finally, let us recognize the built-in bias in free-will research, which is typically rooted in materialistic determinism. Experiments are often designed to falsify the free-will hypothesis. Dennett (2003) provides the philosophical argument that determinism and free will can be compatible.

The origin of intents, choices, and decisions may well arise through either subconscious or conscious mechanisms. In the unified mind of embodied brain, all major acts of will may involve cooperative engagement of both subconscious and conscious minds in the genesis of zombian or free will, or some combination of both. The required neural “machinery” will depend on the nature of willed actions. Simple, well-learned, or habitual tasks, can be a zombian process. Complex or novel tasks may require free-will operation of the conscious mind. Both minds interact and inform each other to varying degrees of what each is doing. Each can guide and influence the actions of the other. In the case of conscious mind, the feedback to subconscious operations also serves a programming function. Providing such programming can even be a free-will intention.
